# Concepts in immuno-oncology: tackling B cell malignancies with CD19-directed bispecific T cell engager therapies

**DOI:** 10.1007/s00277-020-04221-0

**Published:** 2020-08-27

**Authors:** Andreas Viardot, Franco Locatelli, Julia Stieglmaier, Faraz Zaman, Elias Jabbour

**Affiliations:** 1 Department of Internal Medicine III, University Hospital Ulm, Ulm, Germany; 2 Department of Pediatric Hematology and Oncology, IRCCS Bambino Gesù Children’s Hospital, Sapienza, University of Rome, Rome, Italy; 3 Early Development Hematology/Oncology, Amgen Research (Munich) GmbH, Staffelseestraße 2, 81477 Munich, Germany; 4 Amgen Inc., Thousand Oaks, CA USA; 5 Department of Leukemia, The University of Texas M. D. Anderson Cancer Center, Houston, TX USA

**Keywords:** Acute lymphoblastic leukemia, Non-Hodgkin lymphoma, B cell malignancies, Bispecific T cell engager, BiTE molecule, Blinatumomab, CD19

## Abstract

The B cell surface antigen CD19 is a target for treating B cell malignancies, such as B cell precursor acute lymphoblastic leukemia and B cell non-Hodgkin lymphoma. The BiTE® immuno-oncology platform includes blinatumomab, which is approved for relapsed/refractory B cell precursor acute lymphoblastic leukemia and B cell precursor acute lymphoblastic leukemia with minimal residual disease. Blinatumomab is also being evaluated in combination with other agents (tyrosine kinase inhibitors, checkpoint inhibitors, and chemotherapy) in various treatment settings, including frontline protocols. An extended half-life BiTE molecule is also under investigation. Patients receiving blinatumomab may experience cytokine release syndrome and neurotoxicity; however, these events may be less frequent and severe than in patients receiving other CD19-targeted immunotherapies, such as chimeric antigen receptor T cell therapy. We review BiTE technology for treating malignancies that express CD19, analyzing the benefits and limitations of this bispecific T cell engager platform from clinical experience with blinatumomab.

## Introduction

Immuno-oncology therapies, including BiTE® (bispecific T cell engager) molecules, activate the patient’s own immune system to engage with cancer cells. Despite advances in this field, opportunities remain to improve clinical benefit, enhance tolerability, and maximize access. A proven immuno-oncology target for B cell malignancies is CD19, a transmembrane glycoprotein widely and stably expressed on cells of B cell origin. Importantly, CD19 expression is largely limited to B cells, being expressed in most B cell malignancies and some acute myelogenous leukemia [1]. The BiTE molecule blinatumomab (BLINCYTO®, Amgen Inc., Thousand Oaks, CA) and chimeric antigen receptor (CAR) T-cell therapies axicabtagene ciloleucel (Yescarta®, Kite Pharma, Inc., Santa Monica, CA) and tisagenlecleucel (Kymriah™, Novartis Pharmaceuticals Corporation, East Hanover, NJ), all CD19 directed, have been approved for treatment of selected B cell malignancies. Tafasitamab, an anti-CD19 monoclonal antibody in phase 2 studies, also looks promising for treatment of diffuse large B cell lymphoma (DLBCL) [2]. However, primary refractory DLBCL and double hit lymphoma were excluded, making the comparison with other agents more difficult. In addition to anti-CD19 monoclonal antibodies, at the time of this review, there are at least five CD20-CD3 bispecific antibodies under ongoing clinical investigation with promising results in different types of lymphoma. Furthermore, inotuzumab ozogamicin, an anti-CD22 antibody-drug conjugate, has been approved in the EU and US for the treatment of relapsed or refractory (R/R) B cell precursor acute lymphoblastic leukemia (BCP-ALL), providing another treatment option for these patients [3].

Standard-of-care therapy for BCP-ALL includes cytotoxic chemotherapy, corticosteroids, and anti-CD20 antibodies in selected patients; for Philadelphia chromosome-positive (Ph+) ALL, tyrosine kinase inhibitors (TKI) may be included [4–6]. Although in children and young adults, the cure rate is high, the outcome for adults and, in particular, those > 55 years of age is worsened owing to a higher frequency of unfavorable genetic subgroups, a poorer tolerability of chemotherapy regimens, and higher treatment-related mortality [7–11]. Overall, around 50–60% of adults and 15–20% of children with BCP-ALL experience relapse or die from their disease [12–14].

Chemotherapy regimens are also a main therapy for non-Hodgkin lymphoma (NHL). The monoclonal antibody rituximab combined with cyclophosphamide, doxorubicin, vincristine, and prednisone (R-CHOP) is the standard of care for the most frequently diagnosed subtype of NHL, DLBCL. Despite a relatively high number of cures achieved with R-CHOP (~ 50–70%), this treatment is inadequate in 30–40% of patients [15]. Treatment with upfront autologous hematopoietic cell transplant (HSCT) for DLBCL is not considered a standard option, but it may be used with success following chemotherapy failure, increasing event-free and overall survival [16]. However, patients with early relapse (within 12 months after diagnosis) have a poor prognosis regardless of whether any further treatment is given [16, 17]. CAR T cell therapy can confer durable remissions in NHL, specifically DLBCL; however, only phase 2 data are available. Given the relatively low rate of complete remission (CR) after 6 months (40–50%) with CAR T cell therapy [18], there remains a need for more effective and better-tolerated therapies, particularly for high-risk and older patients.

## Mechanism of action for CD19-targeted BiTE therapy

**Fig. 1:**
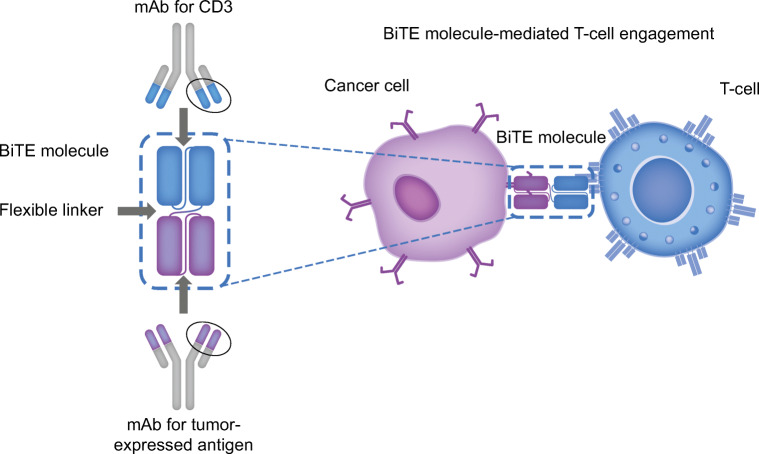
Mechanism of action of BiTE technology. *BiTE* bispecific T cell engager, *CD3* cluster of differentiation 3, *mAb* monoclonal antibody

Therapies based on BiTE technology engage the patient’s own T cells with a tumor-expressed antigen, activating the cytotoxic potential of T cells to eliminate cancer. All BiTE molecules are constructed from one binding domain specific for the CD3 complex presented on T cells, and a second binding domain specific for a target protein expressed by malignant cells. The coupling of these two domains allows BiTE molecules to engage T cells with malignant cells to facilitate malignant cell lysis and initiate a polyclonal T cell response (Fig. 1) [19–21]. By relying on tumor-antigen expression rather than T cell specificity, BiTE technology bypasses the major histocompatibility complex barrier and circumvents a common evasion mechanism of cancer cells [20, 22]. BiTE molecules have been engineered to engage with a variety of malignant cell targets, including B cell maturation antigen, CD33, delta-like protein 3, and prostate-specific membrane antigen.

## Administration, dosing, and safety

### Administration

#### Continuous intravenous infusion

In early phase 1 dose-escalation studies, blinatumomab was administered on a short-term infusion schedule (2–4 h; 1–3 times/week). Unfortunately, no sustained B cell depletion or objective clinical responses were observed [23]. The size of canonical BiTE molecules such as blinatumomab (55 kD) allows for rapid clearance relative to monoclonal antibodies. With a serum half-life of about 2 h, administration by continuous intravenous infusion (cIV) is necessary for sustained drug exposure. Exposure-efficacy analyses show an association between higher blinatumomab steady-state concentrations and a longer duration of survival. The short serum half-life of blinatumomab allows serum levels to be controlled precisely and therefore reduced quickly if needed to manage an adverse event [21]. Blinatumomab can be administered via a portable minipump for cIV, allowing for transition to outpatient treatment [19].

A half-life extended (HLE) CD19 BiTE molecule has been developed to allow greater dosing flexibility with sustained activity. The CD19 HLE BiTE molecule (AMG 562) is composed of CD19- and CD3-binding domains fused to a single chain fragment crystallizable domain to increase the serum half-life. AMG 562 mediates the lysis of CD19-expressing cells at concentrations in the low picomolar range [24]. A phase 1 study investigating the safety and efficacy of AMG 562 in patients with R/R DLBCL, mantle cell lymphoma, or follicular lymphoma has been initiated (NCT03571828) [25].

#### Subcutaneous administration

BiTE molecules have bioavailability after subcutaneous bolus injection [21]. A phase 1b study evaluating the pharmacokinetics/pharmacodynamics of SC administration of blinatumomab in patients with R/R indolent NHL is currently recruiting (NCT02961881) (Table 1).

**Table 1:** Non-pivotal, ongoing, and planned clinical studies on CD19-directed BiTE technology by indication

Indication	Clinical study	Study phase	Study therapy	Completion year
BCP-ALL	Blinatumomab in Adult Patients With Minimal Residual Disease (MRD) of B-precursor Acute Lymphoblastic Leukemia (NCT03109093)	Phase 2	Blinatumomab (AMG103)	Recruiting (2020)
BCP-ALL	Treatment of Older Patients With B-precursor ALL With Sequential Dose Reduced Chemotherapy and Blinatumomab (EWALL-BOLD) (NCT03480438)	Phase 2	Blinatumomab (AMG103) plus SOC chemotherapy	Recruiting (2020)
BCP-ALL	Allogeneic Donor Lymphocyte Infusions Combined With Blinatumomab (DLI-TARGET) (NCT03982992)	Phase 2	Blinatumomab (AMG103) in combination with donor lymphocyte infusion	Recruiting (2020)
r/r B-cell NHL	A Phase II Study Of Blinatumomab For The Treatment Of Relapsed Or Refractory Indolent Non-Hodgkin Lymphoma (NCT02811679)	Phase 2	Blinatumomab (AMG103), autologous stem cell transplant, Carmustine, Etoposide, Cytarabine, Melphalan	Recruiting (2020)
R/R B-cell NHL	A Phase 1b Open-Label Study Investigating the Safety and Pharmacokinetics of Administration of Subcutaneous Blinatumomab for the Treatment of Relapsed/Refractory Indolent Non-Hodgkin's Lymphoma (NCT02961881)	Phase 1b	Blinatumomab (AMG103)	Recruiting (2020)
DLBCL	Blinatumomab Consolidation Post Autologous Stem Cell Transplantation in Patients With Diffuse Large B-Cell Lymphoma (DLBCL) (NCT03072771)	Phase 1	Blinatumomab (AMG103)	Recruiting (2020)

*BCP-ALL* B cell precursor acute lymphoblastic leukemia, *DLBCL* diffuse large B cell lymphoma, *FL* follicular lymphoma, *MCL* mantle cell lymphoma, *NHL* non-Hodgkin lymphoma, *R/R* relapsed or refractory, *SOC* standard-of-care

### Dosing

Dosing of blinatumomab varies by indication and tumor cell burden. Briefly, blinatumomab treatment of BCP-ALL in hematologic complete remission (CR) with persistent minimal residual disease (MRD) consists of one induction cycle (28 days of cIV, then a 14-day treatment-free interval) followed by up to three additional cycles for consolidation [19]. Hospitalization is recommended for the first 3 days of the first cycle and the first 2 days of the second cycle. For R/R BCP-ALL, up to two induction cycles are indicated, followed by consolidation and maintenance cycles; hospitalization is recommended for the first 9 days of the first cycle and the first 2 days of the second cycle [19]. To minimize cytokine release syndrome (CRS), patients are premedicated with corticosteroids, such as dexamethasone, and a step-up blinatumomab dosing regimen in R/R disease is often used, especially in patients with > 25% blasts in the bone marrow [19]. In NHL, a phase 2 study determined that stepwise dosing was tolerable (9 μg/day in week 1, 28 μg/day in week 2, and 112 μg/day for 6 weeks thereafter). Stepwise dose escalation to the target dose is needed to mitigate neurologic events, as two patients treated with a flat dose of 112 μg/day had grade 3 neurologic events. After four treatment-free weeks, a further consolidation cycle in patients with CR, partial response, or stable disease was given. As DLBCL progresses rapidly, stepwise dosing is a limitation that can impact efficacy, and so, approaches that allow patients to receive the target dose without early dropout need to be defined [26].

### Safety

Well-documented risks associated with blinatumomab are CRS and neurologic events (recently classified as immune effector cell-associated neurologic syndrome), which are mostly manageable, and medication errors [27–29]. Strategies to mitigate these risks include pretreatment with corticosteroids, dose adjustments, and preparation guidelines; research is ongoing to aid in the prevention of these risks [27, 30]. Other adverse events after administration of blinatumomab have been reported, including tumor lysis syndrome, cytopenias, pyrexia, and anemia [31]. CAR T cell therapies have been associated with severe CRS, as well as neurologic events, infections, hemophagocytosis, and cytopenia [29, 32, 33].

CRS can result following treatment if hyperactivation of immune cells occurs. With increasing experience in the use of CAR T cell therapies, CRS grading has been redefined several times recently, leading to the development of the Penn grading scale, which assigns grades to guide CRS management distinguishing between mild, moderate, severe, and life-threatening CRS [28, 34]. Blinatumomab trials generally used common terminology for adverse events (Common Terminology Criteria for Adverse Events grading scale; CTCAE), but as noted, this has been deemed inadequate for use with cellular therapy [34]. Around 5% of patients with R/R BCP-ALL or MRD-positive BCP-ALL experienced a serious CRS event (CTCAE grade ≥ 3) [27, 35]. In B cell lymphoma phase 2 studies, grade ≥ 3 CRS was detected in only 1 (2%) of patients; no grade ≥ 3 (CTCAE) CRS events were reported in the other studies [26, 36, 37]. Rates of CRS events were higher for patients following CAR T cell therapies. Grade ≥ 3 (CTCAE) CRS events were reported in 13% of adults with B cell lymphoma receiving axicabtagene ciloleucel [32, 38]; 22% of adults with R/R DLBCL receiving tisagenlecleucel (Penn grading scale) [39]; and 47% of pediatric and young adult patients with R/R ALL receiving tisagenlecleucel (Penn grading scale) [40]. Predictive biomarkers are being sought to better understand CRS biology and to guide therapy [41].

Premedication with corticosteroids before starting blinatumomab treatment is recommended to mitigate CRS [19]. The interleukin-6 receptor antagonist (tocilizumab) has been approved to treat severe or life-threatening CRS in patients receiving CAR T cell therapy either alone or in combination with corticosteroids [42]. Tocilizumab use is possible but infrequent in blinatumomab trials, as CRS can usually be controlled by corticosteroids or an interruption of infusion, which is not possible with CAR T cell therapy [19, 42, 43].

Neurotoxicity is associated with blinatumomab and CAR T cell therapy targeting CD19. During phase 2 studies of blinatumomab in patients with BCP-ALL, up to 53% experienced neurologic events of any grade, and up to 13% had grade ≥ 3/4, with no associated deaths [44, 45]. In DLBCL, 22% experienced grade 3/4 neurotoxicity [26]. During phase 1 and 2 CAR T cell trials, 38% experienced neurotoxicity at grades 1/2 and 5% at ≥ 3 in the former, and up to 40% had any grade and 13% had grade 3 in the latter; 2 patients had unresolved grade 3 neurologic events at the time of death [40, 46]. Due to its high clearance rate, interruption of blinatumomab treatment is sufficient to resolve most neurologic events, although some resulted in treatment discontinuation. While the exact mechanism of CD19-mediated neurotoxicity is unclear, adhesion of T cells to endothelial cells may be involved [47].

### Impact of tumor burden

Observations in clinical trials suggest that tumor burden has an impact on the efficacy and safety of CD19 BiTE therapies. Subgroup analyses from previous trials in patients with Ph-BCP-ALL showed that the greatest overall survival (OS) and remission rates were observed in populations with < 50% bone marrow blasts treated with blinatumomab [48]. Currently, there are several studies in progress or planned to assess the use of blinatumomab in patients who have received debulking chemotherapy (NCT03023878, NCT03931642) (Table 1).

## Efficacy of blinatumomab in BCP-ALL (Table 2)

### Adults with BCP-ALL in CR with MRD

The complete elimination of molecular disease is desirable, as patients with BCP-ALL in hematologic CR with MRD are at high risk of relapse. In the phase 2 BLAST study (NCT01207388) conducted in adults with BCP-ALL in CR with MRD (≥ 10^−3^), complete MRD response (no target amplification with a minimum sensitivity of 10^−4^) was achieved in 78% (95% confidence interval, 69–85%) of patients within one blinatumomab treatment cycle. Median relapse-free survival was 23.6 months versus 5.7 months (*p* = 0.002) and median OS was 38.9 months versus 12.5 months (*p* = 0.002) in patients who did or did not achieve complete MRD response within one cycle of treatment, respectively [44]. In the final 5-year follow-up analysis, median OS was not reached among patients with a complete MRD response in cycle 1, indicating cure in these patients [49]. As the BLAST study was a single-arm study, an analysis of hematological relapse-free survival using “historical-comparator” control data was performed to further substantiate the benefit of blinatumomab [50]. The results of this single-arm study led to blinatumomab receiving approval for use in patients with BCP-ALL in CR with MRD.

**Table 2:** Key blinatumomab clinical outcomes

Pivotal study	Study population	Primary outcome	Other key outcomes
NCT02013167 (TOWER) [43]	R/R Ph– BCP-ALL (Adult)	Median OS: 7.7 months (95% CI, 5.6–9.6 months)	CR within 12 weeks of treatment initiation: 91/267 (34%) (95% CI, 28.0–39.5%; *p* < 0.001) CRh within 12 weeks of treatment initiation: 119/267 (44%) (95% CI, 37.9–50.0%; *p* < 0.001) EFS (6-month estimate): 31% MRD remission (defined as an MRD level below 0.0001): 76% Adverse events (grade ≥ 3): 231/267 (87%)
NCT01466179 (Study MT103-211) [40]	R/R Ph– BCP-ALL (Adult)	CR or CRh: 81/189 (43%) (95% CI, 36–50%) within the first two cycles of treatment	Median RFS in patients with CR/CRh: 5.9 months (95% CI, 4.8–8.3 months) Median OS: 6.1 months (95% CI, 4.2–7.5 months) alloHSCT after blinatumomab-induced remission: 32/81 (40%) 100-day mortality following alloHSCT: 11% (95% CI, 0–23%) MRD response: 60/73 (82%) (95% CI, 72–90%) Adverse events (grade ≥ 3): 71 (38%)
NCT01207388 (BLAST) [39]	MRD-positive BCP-ALL (Adult)	Complete MRD response: 88/113 (78%) patients after one cycle of treatment	Median OS: 36.5 months (95% CI, 19.8 months to not estimable) Median RFS: 18.9 months (95% CI, 12.3–35.2 months) Duration of hematologic remission: not reached
NCT02000427 (ALCANTARA) [49]	R/R Ph+ BCP-ALL (Adult)	CR or CRh: 16/45 (36%) (95% CI, 22–51%) within the first two cycles of treatment	Complete MRD response: 14/16 (88%) (95% CI, 62–98%) during the first two cycles of treatment Median RFS: 6.7 months (95% CI, 4.4 months to not estimable) Median OS: 7.1 months (95% CI, 5.6 months to not estimable) alloHSCT after blinatumomab-induced remission: 4/16 (25%) (95% CI, 7–52%) Adverse events (grade ≥ 3): 37/45 (82%)
NCT01471782 (Study MT103-205) [29]	R/R BCP-ALL (Pediatric)	Maximum-tolerated dosage: 15 mg/m^2^/day CR: 27/70 (39%) (95% CI, 27–51%)	Median RFS in responders (*n* = 27): 4.4 months (95% CI, 2.3–7.6 months) Median OS (*n* = 70): 7.5 months (95% CI, 4.0–11.8 months) alloHSCT after blinatumomab treatment: 24/70 (34%) Complete MRD response (< 10^–4^): 14/27 (52%) (95% CI, 32–71%) Adverse events (grade ≥ 3): 61 (87%)

*alloHSCT* allogeneic hematopoietic stem cell transplant, *CI* confidence interval, *CR* complete remission, *CRh* complete remission with partial hematologic recovery of peripheral blood counts, *EFS* event-free survival, *MRD* minimal residual disease, *OS* overall survival, *RFS* relapse-free survival, *R/R BCP-ALL* relapsed or refractory B cell precursor acute lymphoblastic leukemia, *R/R Ph+ BCP-ALL* relapsed or refractory Philadelphia chromosome-positive B cell precursor acute lymphoblastic leukemia, *R/R Ph– BCP-ALL* relapsed or refractory Philadelphia chromosome-negative B cell precursor acute lymphoblastic leukemia

### Adults with R/R BCP-ALL

The multinational, randomized, phase 3 TOWER study (NCT02013167) examined the outcomes of heavily pretreated adults with Philadelphia chromosome-negative (Ph−) R/R BCP-ALL who were given either blinatumomab or standard-of-care chemotherapy (Table 2) [48]. Patients receiving blinatumomab experienced a deep and durable response to treatment and significantly longer OS compared with patients receiving chemotherapy. Notably, the trial was stopped early because of the benefit to OS seen in the blinatumomab cohort [48]. Blinatumomab, beneficial for OS in first or later salvage, was particularly effective in first salvage, where it more than doubled median survival relative to chemotherapy [51]. Salvage status drove survival in responders regardless of subsequent allogeneic stem cell transplantation [52]. Blinatumomab also provided clinically meaningful benefits in health-related quality of life [53].

The presence of Ph+ is associated with a poor prognosis in ALL and is commonly seen in older patients. TKIs combined with chemotherapy are an established frontline therapy for Ph+ BCP-ALL in adults. The pivotal phase 2 ALCANTARA study (NCT02000427) was a single-arm trial with blinatumomab in patients with Ph+ BCP-ALL who were refractory/R or intolerant to second-generation or later TKIs and/or to imatinib [54, 55]. ALCANTARA demonstrated CR or CR with partial hematologic recovery in 16/45 (36%) patients and blinatumomab was also highly effective at eliminating detectable MRD in patients with R/R Ph+ BCP-ALL, as 12/14 (86%) responders achieved a complete MRD response [55].

### Children and adolescents with R/R BCP-ALL

Blinatumomab is highly effective in high-risk pediatric patients with R/R BCP-ALL. Enrollment in two phase 3 trials conducted in children with high-risk first relapse BCP-ALL was terminated early due to encouraging efficacy in the blinatumomab arm [56]. These findings corroborate earlier studies; in a phase 1/2 trial conducted in children with CD19-positive BCP-ALL that were in second or later relapse, any relapse after allogeneic HSCT, or refractory to other treatments and with > 25% bone marrow blasts at screening, complete hematologic responses were observed in 27 of the 70 patients (median [range] age of 8 [< 1–17] years) who received the recommended dose of blinatumomab. Fourteen of these 27 children (52%) achieved a complete MRD response [35]. RIALTO (NCT02187354), conducted in children in the same disease phase, corroborated the evidence of efficacy and safety of blinatumomab in the pediatric population. Of 98 children with ≥ 5% blasts, 58 (59%) achieved CR, while among the 12 with < 5% blasts but with MRD level ≥ 10^−3^, 11 (92%) achieved MRD response [57].

### BCP-ALL subtypes

Ph-like BCP-ALL is a subtype of BCP-ALL with a gene expression profile like Ph+ BCP-ALL. It appears to be most prevalent in adolescents, young adults, and adults > 40 years of age. Regardless of age, patients with select Ph-like BCP-ALL subtypes have a higher rate of relapse and lower OS and are more likely to have MRD post-chemotherapy [58, 59]. It is theorized that therapies with proven efficacy against Ph+ disease and MRD may be effective in these patients, although additional evidence is needed [59]. The fusion gene TCF3-HLF-positive leukemia, caused by the chromosomal translocation t(17;19), represents a rare cytogenetic subtype of BCP-ALL occurring in children and young adults. It is associated with poor outcomes, despite treatment intensification and HSCT [60, 61]. There is promising evidence that blinatumomab might help improve the quality of remission as a bridge to HSCT [61].

### Combination and sequential therapies

As some patients relapse following blinatumomab monotherapy, combination therapy regimens with blinatumomab are being investigated to improve outcomes. Expression of immune checkpoint proteins, including programmed cell death-1 (PD-1), PD-ligand 1 (PD-L1), and cytotoxic T-lymphocyte-associated protein 4 (CTLA-4), can contribute to resistance, suggesting that combination therapy, including immune checkpoint inhibition, may be effective [62]. Additional strategies with blinatumomab include combination with low-dose chemotherapy to reduce the intensity of chemotherapy and associated adverse events, and with TKIs to avoid chemotherapy completely (Table 3).

**Table 3:** Summary of different therapies being explored in combination with blinatumomab

Study	Study phase	Indication	Other study drug	Class of other study drug
D-ALBA Frontline Sequential Dasatinib and Blinatumomab in Adult Philadelphia Positive Acute Lymphoblastic Leukemia [61]	Phase 2	(Ph+) BCP-ALL, (Ph-like BCP-ALL)	Dasatinib	TKI
Blinatumomab and Combination Chemotherapy or Dasatinib, Prednisone, and Blinatumomab in Treating Older Patients With Acute Lymphoblastic Leukemia	Phase 2	(R/R Ph+ and Ph−) BCP-ALL	Dasatinib, prednisone, vincristine, methotrexate, 6-mercaptopurine	TKI plus chemotherapy
Relapsed Philadelphia chromosome-positive pre-B-ALL after CD19-directed CAR-T cell therapy successfully treated with combination of blinatumomab and ponatinib	N/A	(R/R Ph+) BCP-ALL	Ponatinib	TKI
Chemoimmunotherapy with inotuzumab ozogamicin combined with mini-hyper-CVD, with or without blinatumomab in patients with Philadelphia chromosome-negative acute lymphoblastic leukemia in first salvage	N/A	R/R (Ph−) ALL	Inotuzumab ozogamicin	Monoclonal antibody
Blinatumomab in combination with immune checkpoint inhibitors of PD-1 and CTLA-4 in adult patients with relapsed/refractory (R/R) CD19 positive B-cell acute lymphoblastic leukemia (ALL) [57]	Phase 1	R/R BCP-ALL	Nivolumab/ipilimumab	Monoclonal antibody (checkpoint inhibitors)
A phase I/II study of blinatumomab in combination with pembrolizumab for adults with relapsed refractory B-lineage acute lymphoblastic leukemia: University of California Hematologic Malignancies Consortium Study 1504 [58]	Phase 1/2	R/R BCP-ALL	Pembrolizumab	Humanized antibody (checkpoint inhibitor)
Treatment Protocol for Children and Adolescents With Acute Lymphoblastic Leukemia - AIEOP-BFM ALL 2017 (NCT03643276)	Phase 3	BCP-ALL	Multiple	Chemotherapy/other

*BCP-ALL* B cell precursor acute lymphoblastic leukemia, *Ph+ BCP-ALL* Philadelphia chromosome-positive B cell precursor acute lymphoblastic leukemia, *R/R BCP-ALL* relapsed or refractory B cell precursor acute lymphoblastic leukemia, *R/R Ph+ BCP-ALL* relapsed or refractory Philadelphia chromosome-positive B cell precursor acute lymphoblastic leukemia, *R/R Ph– BCP-ALL* relapsed or refractory Philadelphia chromosome-negative B cell precursor acute lymphoblastic leukemia, *TKI* tyrosine kinase inhibitor

#### Blinatumomab plus PD-1 and CTLA-4 inhibitors in R/R BCP-ALL

The combination of blinatumomab with nivolumab (anti-PD-1; OPDIVO®, Bristol-Myers Squibb Company, Princeton, NJ) in patients with R/R ALL is being investigated in a phase 1 study, with later intensification to include ipilimumab (anti-CTLA-4; YERVOY®, Bristol-Myers Squibb Company, Princeton, NJ). Preliminary findings suggest that blinatumomab plus nivolumab in R/R ALL is feasible with acceptable toxicity. The MRD complete response rate was 80% despite patients being heavily pretreated [63].

A phase 1/2 study is being conducted to determine whether blinatumomab with pembrolizumab (anti-PD-1) improves efficacy versus blinatumomab monotherapy in adults with R/R BCP-ALL with a high percentage of bone marrow blasts (> 50% lymphoblasts) (NCT03512405) [62]. Although mechanisms of resistance to blinatumomab are not well understood, the upregulation of checkpoint proteins could inhibit T cell function to cause resistance [62]. The addition of pembrolizumab to blinatumomab may increase the activity of blinatumomab, potentially leading to more patients achieving deep and durable remissions [62].

#### Blinatumomab plus TKIs in Ph+ R/R BCP-ALL

Limited data suggest that the combination of blinatumomab and TKIs may provide additional benefit in patients with Ph+ BCP-ALL. In a retrospective study of 12 patients, 9 with R/R BCP-ALL and 3 with chronic myeloid leukemia in blast crisis, treatment was with blinatumomab plus TKIs (ponatinib [*n* = 8]; dasatinib [*n* = 3]; bosutinib [*n* = 1]). The complete hematologic, cytogenetic, and molecular response rates were 50% (3/6), 71% (5/7), and 75% (9/12), respectively [64]. In another study of 11 patients receiving blinatumomab in combination with TKI therapy, 89% (8/9) of patients with MRD achieved complete molecular response [65]. An ongoing phase 2 study (NCT02744768) is evaluating the potential for a chemotherapy-free induction-consolidation frontline regimen of dasatinib combined with blinatumomab in obtaining an MRD response in patients with newly diagnosed Ph+ BCP-ALL. Preliminary results are promising, with 54% (19/35) of patients achieving a molecular response (10/19 with complete molecular remission) after two cycles of treatment and increasing after the third (68%) and fourth (80%) cycles. To date, the 12-month OS and disease-free survival rates are 96% and 92%, respectively [66].

#### Blinatumomab use in frontline BCP-ALL therapy in older patients

Survival is poor for older patients with BCP-ALL. In a phase 2 study of newly diagnosed patients ≥ 60 years of age, blinatumomab was added sequentially to mini-hyperfractionated cyclophosphamide, vincristine, dexamethasone (mini-HCVD), and inotuzumab, and survival was significantly improved relative to historical hyperfractionated cyclophosphamide, vincristine, doxorubicin, and dexamethasone therapy with a 3-year OS rate of 54% (median not reached) versus 32% (median, 16 months) (*P* = 0.003) [67]. Blinatumomab was added to improve efficacy and optimize safety, while minimizing the risk of liver veno-occlusive disease [68].

In a phase 2 study of newly diagnosed high-risk patients > 65 years of age, blinatumomab followed by prednisone, vincristine, methotrexate, 6-mercaptopurine maintenance was effective with a 66% (19/29) response rate; 12 of 13 responders with MRD data achieved an MRD complete response after one cycle of blinatumomab [69].

#### Blinatumomab use in first-salvage, mini-HCVD, and inotuzumab in BCP-ALL therapy

In a similar phase 2 study of young (median age of 39 years) patients (*n* = 48) with R/R BCP-ALL in first salvage, better outcomes were achieved with the combination of mini-HCVD and inotuzumab with or without blinatumomab than intensive chemotherapy or inotuzumab alone [70].

## Efficacy of CD19-targeted BiTE molecules in B cell NHL

### Blinatumomab

The initial phase 1 dose-escalation study of blinatumomab monotherapy in heavily pretreated patients with R/R NHL had positive results, with an overall response rate (ORR) of 69% at or below the target dose of 60 μg/m^2^/day; the ORR was 55% for patients with the DLBCL subtype [71].

In a phase 2 study focusing on R/R DLBCL, patients who received blinatumomab for at least 1 week at the target dose or stopped treatment sooner due to disease progression were evaluated (*n* = 21 [84%]). Of these patients, an ORR was observed in 9 (43%) and a complete response in 4 (19%). As patients had received a median of three prior lines of therapy, these results were encouraging [26].

A phase 2/3 study evaluated adult patients (*n* = 41) with highly aggressive R/R B cell NHL. Most patients had progressive disease (27/41 [66%]) and were refractory to (28/41 [68%]) or relapsed (13/41 [32%]) after frontline therapy. An ORR of 37% was observed in 15 patients and 9 (22%) patients had a complete response (NCT02910063) [36].

Additional studies of blinatumomab for newly diagnosed high-risk DLBCL (NCT03023878), high-risk DLBCL post-autologous HSCT (NCT03072771), and in combination with pembrolizumab for R/R DLBCL (NCT03340766) are ongoing.

Positive results have also been observed in case studies of blinatumomab treatment for R/R Burkitt’s lymphoma. Three patients who were refractory to first-line chemotherapy were treated with blinatumomab, one of whom reached CR after two cycles of treatment. This patient later relapsed with Burkitt’s lymphoma following autologous HSCT; however, re-treatment with blinatumomab induced a second CR after one cycle of treatment and the patient received six additional cycles for consolidation/maintenance. This patient is now 18 months in continuous CR with complete MRD response, demonstrating that blinatumomab shows activity in patients with R/R Burkitt’s leukemia and lymphoma [72].

## Modes of resistance with CD19-directed therapies

### Antigen escape

The loss of expression of target antigens by cancer cells is a common escape mechanism that occurs following therapies. About 35% (determined by flow cytometry on leukemic blasts) of R/R ALL blinatumomab responders had dim or negative CD19 expression, although following blinatumomab failure in patients with R/R ALL, a minimal change in the level of CD19 expression was reported [73, 74]. Following CAR T-cell therapy for ALL, rates of CD19 negativity appear to be approximately 10–20% [75]. Mutations with an effect on CD19 protein were identified in less than one-third of CD19-negative relapsed patients with BCP-ALL treated with blinatumomab in contrast to truncating CD19 mutations in 12/12 patients with CD19-negative relapse after CAR T cell therapy [76]. This difference could be due to a stronger selection pressure of CAR T cell therapy versus blinatumomab [77]. As most patients with ALL (92%) stay CD19 positive following blinatumomab, they would potentially benefit from subsequent CD19 immuno-oncology therapy [74].

### Dysregulated immune checkpoint pathways and T cell exhaustion

Induced T cell dysfunction may be a potential mechanism of resistance to BiTE-mediated therapy as a preclinical study identified upregulation of the checkpoint inhibitor PD-1 in malignant cells exposed to blinatumomab, and in vivo upregulation of PD-1 and PD-L1 was observed in an ALL patient who had received blinatumomab [78]. The in vitro addition of PD-1 with or without CTLA-4 inhibitors with blinatumomab to BCP-ALL blasts and peripheral blood mononuclear cells led to increased T cell proliferation and enhanced blinatumomab-mediated cytotoxicity [79]. This suggests that blockade of co-inhibitory pathways could prevent resistance to blinatumomab in some patients and provides a rationale for the combination studies of blinatumomab and PD-1 inhibitors, which are currently ongoing.

The presence of regulatory T cells (Treg) might also impair blinatumomab responses; in patients with R/R BCP-ALL, it was found that Treg enumeration could identify 100% of responders and exclude 70% of nonresponders [80]. Depletion of Tregs restored the proliferation of T cells and, therefore, may convert blinatumomab nonresponder ALL patients to responders [80].

### Lineage switch

Lineage switch, a conversion between lymphoid and myeloid cell lineages during therapy resulting in the loss of CD19 expression, has been observed following CD19-directed CAR T cell therapy of ALL and blinatumomab treatment [81–83]. A high proportion of infant leukemias is characterized by mixed-lineage leukemia involving the KMT2A translocation gene; this rearrangement is seen in 5% of childhood cases, but in 70–80% of ALL infants [84]. Lineage switch may also be seen in patients with Ph+ ALL [85].

## Health economics and outcomes of CD19 therapies

The benefit of blinatumomab is that the deterioration in most health-related quality of life measures relevant to oncology is delayed compared with chemotherapy [53]. A US healthcare cost-benefit analysis using phase 3 data from TOWER estimated that blinatumomab added 1.92 life years and 1.64 quality-adjusted life years (QALY) compared with chemotherapy [48, 86]. Cost-effectiveness was particularly favorable for patients who had received no prior salvage therapy. The incremental cost-effectiveness ratio for blinatumomab versus chemotherapy was estimated to be $110,108/QALY gained. Therefore, compared with chemotherapy, blinatumomab was deemed a cost-effective treatment option for adults with R/R Ph– BCP-ALL at an incremental cost-effectiveness ratio threshold of $150,000/QALY gained [86].

Both blinatumomab and the commercial CAR T cell preparations have considerable costs. For BCP-ALL, tisagenlecleucel costs start at $475,000, whereas blinatumomab costs $178,000 (two treatment cycles), but costs will depend on the number of cycles needed [87, 88]. These figures do not factor in the length of time required in the hospital, so the true cost for both may be higher, although if durable responses are achieved without HSCT, they might be lower [89]. As a highly individualized therapy, CAR T cell therapy faces a manufacturing burden that affects the monetary cost of treatment and can cost the patient valuable time [88]. The process, from collection of patients’ T cells, reengineering the T cells in a laboratory, to infusion of modified T cells/CAR T cells in the patient can take up to 4 weeks [90]. In contrast, BiTE molecules are an off-the-shelf therapy with immediate availability to any patient. As noted, the short half-life of blinatumomab means adverse events can be managed more easily and quickly compared with CAR T cell therapy. Once administered, the CAR T cells expand in vivo and can persist for years [91].

A summary of the characteristics of BiTE and CAR T cell technologies is listed in Table 4. Both provide strong T cell immune responses against several tumor types. However, in addition to the above, BiTE molecules may provide some potential advantages over CAR T cell therapy, including higher effectiveness in R/R ALL first salvage versus chemotherapy, defined dosing, and knowledge of their pharmacokinetics [51, 92]. Both CAR T cell therapy and BiTE therapy may promote the occurrence of CRS and neurological adverse effects, although these conditions are more easily managed with dose interruptions when elicited by treatment with a BiTE molecule [29, 33]. More specific shared limitations include dependence on autologous “exhausted” T cells in heavily treated patients, and mechanisms of failure, including immunoediting leading to loss of target antigen, lineage switch, and activation of the programmed death/ligand (PD1/PD-L1) axis [92–95]. CAR-T approach does have some advantages over BiTE therapies, not least applying CAR T cell technology to other immune cells such as natural killer (NK) cells and the use of allogenic T cells that have not been exhausted [96]. All of these factors need to be considered when deciding on the optimal therapy for each individual patient [97].

**Table 4:** Characteristics of blinatumomab and CAR T cell technologies

	BiTE (bispecific T cell engager) technology	Chimeric antigen replacement (CAR) T cell therapy
Structure	A recombinant protein comprising two linked single-chain variable fragment capable of binding to the specified tumor antigen and CD3 on T cell	A construct encoding an scFv against the specified tumor antigen (i.e., CD19 and co-stimulatory domain)
Availability	“Off-the-shelf” product; available immediately to any patient	Requires manufacturing per each individual ~ 4 weeks [84]
Persistence	Short half-life; requires one induction cycle (28 days) of treatment followed by three cycles for consolidation [17, 19]	One infusion, expansion in vivo, may persist for years
Clinical use	Pretreatment with dexamethasone or prednisone required to manage CRS [17]	Pretreatment lymphodepleting regimen required to enhance CAR T-cell expansion and efficacy and tocilizumab must be available to manage CRS [32]
Efficacy	BCP-ALL: see Table 2 R/R DLBCL: ORR, 43% (9/21); median PFS, 3.7 months (95% CI, 1.4–7.7); CR rate, 19% (4/21) [24] R/R NHL: CMR, 22% (9/41); ORR, 37% (15/41) [30]	*Tisagenlecleucel* R/R BCP-ALL (pediatric to young adult): overall remission rate, 81% (61/75); 66% on an intention-to-treat analysis [35] R/R DLBCL: ORR, 52% (48/93) [34] *Axicabtagene ciloleucel* R/R DLBCL: ORR, 82% (63/77) [33]
Toxicity	CRS, neurotoxicity	CRS, neurotoxicity, HLH

*CI* confidence interval, *CMR* complete metabolic response, *CR* complete remission, *CRS* cytokine release syndrome, *MRD* minimal residual disease, *ORR* overall response rate/objective response rate, *NHL* non-Hodgkin lymphoma, *R/R BCP-ALL* relapsed or refractory B cell precursor acute lymphoblastic leukemia, *R/R DLBCL* relapsed or refractory diffuse large B cell lymphoma, *HLH* hemophagocytic lymphohistiocytosis

CAR T cell therapy has shown high complete response rates in some B cell malignancies including NHL, chronic lymphocytic leukemia, and ALL; in addition, it has changed the treatment model for DLBCL, although around a half of patients will not achieve CR or will relapse after therapy [18]. High relapse frequency is seen across all indications [18]. Blinatumomab has shown higher rates of overall survival and hematologic remission than chemotherapy in ALL and has promise in treating NHL [92]. However, it also shows significant relapse rates. In a study in patients with R/R ALL who underwent allogenic HSCT after treatment with blinatumomab, it was found that transplantation reduced the risk of death by 55% and 46% in blinatumomab and SOC groups, respectively [52]. The best outcomes were in patients with no prior salvage therapy and MRD responses to blinatumomab regardless of on-study HSCT status [52]. Effectiveness in terms of durable remission following post CAR T allogenic HSCT seems positive in several studies investigating treatment for R/R ALL [91, 98, 99]. In a clinical trial conducted in pediatric patients with first relapse ALL (AALL1331; NCT02101853), standard salvage treatment was compared with blinatumomab consolidation after an obligate first salvage chemotherapy. The toxicity was significantly reduced after blinatumomab, MRD response was higher, and allogeneic transplant was performed in a significantly higher proportion of patients in the blinatumomab arm resulting in a 20% difference in overall survival [100]. These results were confirmed in a parallel, randomized clinical trial run in children with high-risk first relapse ALL comparing a block of conventional chemotherapy with blinatumomab as consolidation therapy before HSCT (NCT02393859). Longer-term follow-up and additional studies of both BiTE and CAR T cell responders will be helpful in better defining their role in different B cell malignancies.

## Conclusions

The CD19 BiTE therapy blinatumomab has validated the BiTE immuno-oncology platform and raised B-ALL treatment standards, with the first approval of any drug to treat MRD. SC administration will add versatility and patient convenience. Some limitations need to be addressed; additional investigation is needed to understand the impact of stepwise dosing on the efficacy of blinatumomab treatment for DLBCL and to help mitigate further the occurrence of adverse events such as CRS and neurotoxicity, while relatively frequent relapse may affect potential monotherapeutic use if not overcome. CD19 BiTE molecules have antitumor activity as monotherapy and the potential for enhanced activity in combination with other treatments. The off-the-shelf BiTE therapies are cost effective, improve quality of life relative to chemotherapy, and have been used in thousands of patients. BiTE molecules such as blinatumomab continue to generate evidence of a favorable benefit/risk profile across treatment lines and tumor types.
